# Hydrogen-Releasing Micromaterial Dressings: Promoting Wound Healing by Modulating Extracellular Matrix Accumulation Through Wnt/β-Catenin and TGF-β/Smad Pathways

**DOI:** 10.3390/pharmaceutics17030279

**Published:** 2025-02-20

**Authors:** Pengxiang Zhao, Yufei Li, Boyuan Guo, Ziyi Liu, Xujuan Zhang, Mengyu Liu, Xuemei Ma

**Affiliations:** College of Chemistry and Life Sciences, Beijing University of Technology, Beijing 100124, China; zpx@bjut.edu.cn (P.Z.); 13716390418@163.com (Y.L.); 18311481558@163.com (B.G.); 18803296354@163.com (Z.L.); jyzhangxj@bjut.edu.cn (X.Z.); mengyu@bjut.edu.cn (M.L.)

**Keywords:** wound healing, hydrogen-releasing micromaterial, extracellular matrix deposition, angiogenesis, molecular mechanism

## Abstract

**Background**: Wound healing is a complex and intricate biological process that involves multiple systems within the body and initiates a series of highly coordinated responses to repair damage and restore integrity and functionality. We previously identified that breathing hydrogen can significantly inhibit early inflammation, activate autologous stem cells, and promote the accumulation of extracellular matrix (ECM). However, the broader functions and downstream targets of hydrogen-induced ECM accumulation and tissue remodeling are unknown in the wound-healing process. **Methods**: Consequently, this thesis developed a hydrogen sustained-release dressing based on a micro storage material and reveals the mechanism of hydrogen in treating wound healing. Upon encapsulating the hydrogen storage materials, magnesium (Mg), and ammonia borane (AB), we found that SiO_2_@Mg exhibits superior sustained-release performance, while SiO_2_@AB demonstrates a higher hydrogen storage capacity. We used a *C57/BL6* mouse full-thickness skin defect wound model to analyze and compare different hydrogen dressings. **Results**: It was identified that hydrogen dressings can significantly improve the healing rate of wounds by promoting epithelialization, angiogenesis, and collagen accumulation in wound tissue, and that the effect of slow-release dressings is better than of non-slow-release dressings. We also found that hydrogen dressing can promote transcriptome-level expression related to cell proliferation and differentiation and ECM accumulation, mainly through the *Wnt1/β-catenin* pathway and *TGF-β1/Smad2* pathway. **Conclusions**: Overall, these results provide a novel insight into the field of hydrogen treatment and wound healing.

## 1. Introduction

The wound-healing process, initiated by skin injury, can be broadly categorized into three distinct stages: the inflammatory stage, the granulation tissue formation stage, and the matrix remodeling stage [1]. This healing response ultimately leads to the reconstruction of the original tissue.

During wound healing, granulation tissue serves as a provisional biological scaffold that fills tissue defects and facilitates connective tissue regeneration [2]. This dynamic tissue is characterized by the activation of mesenchymal cells which undergo extensive proliferation and orchestrate extracellular matrix (ECM) synthesis [3]. The formative phase involves coordinated proliferation and the migration of multiple cell types to the injury site [4]. Key growth factors secreted by immune cells—particularly transforming growth factor-β (*TGF-β*), vascular endothelial growth factor (*VEGF*), and fibroblast growth factor (*FGF*)—create a biochemical milieu that stimulates keratinocyte and fibroblast proliferation [5]. These cellular interactions, combined with functional protein signaling, establish a provisional ECM network that supports subsequent angiogenesis [6]. The emerging granulation tissue architecture, comprising neovascularization, macrophage populations, and nascent ECM components, provides the structural foundation for epithelial regeneration. A critical phenotypic transition occurs as keratinocyte-mediated signaling induces fibroblast differentiation into contractile myofibroblasts, which actively mediate wound contraction and closure [7]. During the remodeling phase, activated wound cells undergo programmed deactivation while macrophages and fibroblasts gradually dissipate from the healed tissue, ultimately yielding a collagen-rich ECM with sparse cellularity [8,9]. The final tissue restoration involves epithelial–mesenchymal interactions that ensure cutaneous integrity and biomechanical stability [10]. Macrophage-derived and fibroblast-secreted matrix metalloproteinases (*MMPs*) mediate progressive ECM remodeling, during which the initial type III collagen deposition is systematically replaced by stronger type I collagen fibrils, enhancing the tissue’s structural resilience [11].

Impaired wound healing frequently leads to complex clinical sequelae such as chronic ulcers and pressure injuries. The transition to chronic non-healing wounds typically results from multifactorial interactions, including persistent hypoxia, microbial colonization, and growth factor signaling dysregulation. These pathological conditions maintain a protracted inflammatory phase while impairing granulation tissue development [12,13], creating therapeutic challenges due to ambiguous pathogenic mechanisms and extended treatment timelines. Current wound management strategies encompass surgical intervention, topical biologics/pharmaceuticals, and advanced dressing technologies. Among emerging alternatives, hydrogen-based therapies exhibit distinct biomedical advantages. The unique physicochemical properties of molecular hydrogen—including its small molecular size enabling membrane permeability, non-toxic metabolic byproduct (water), and versatile delivery methods (oral, inhalational, and parenteral administration)—position it as an ideal therapeutic candidate. Hydrogen has demonstrated a positive therapeutic effect on UV-induced skin damage [14]. Local applications of hydrogen-rich water can prevent skin inflammation by inhibiting the expression of *MMP-1*, *IL-6*, *IL-1β*, and other mRNAs induced by UV exposure, thereby regulating skin function. Topical hydrogen-rich water also prevents UV-induced skin inflammation and modulates both intrinsic aging and photoaging processes [15]. Hydrogen inhalation mitigates radiation dermatitis severity and accelerates healing through redox homeostasis restoration [16]. In deep burn models, hydrogen facilitates early wound resolution via oxidative stress reduction, apoptosis inhibition, and *Akt/NF-κB* pathway activation, potentially regulating inflammatory cytokine networks [17]. Additionally, oral hydrogen-rich water administration activates the *Nrf2*-mediated antioxidant response in rodent models, synergistically promoting epithelial regeneration [18]. In summary, hydrogen accelerates healing through varied biological modulation, and the pleiotropic effects address multiple pathological aspects of chronic wounds, offering a paradigm shift in regenerative therapy.

In this study, two materials with strong hydrogen storage capabilities, magnesium (Mg) and ammonia borane (AB), were selected as raw materials for hydrogen release, and a new type of hydrogen-releasing dressing was designed. Magnesium (Mg, 41.7 mmol/g) and ammonia borane (AB, 326.5 mmol/g) exhibit strong hydrogen storage capacities. However, the rate of hydrogen release from Mg and AB is too fast, leading to the generation of hydrogen bubbles. These bubbles pose a potential risk of embolism, which could interfere with tissue repair and cause harm to the organism. Therefore, it is essential to achieve slow and sustained Mg and AB degradation for controlled hydrogen production. The hydrophobic SiO_2_ shells can impede water penetration into the inner layer of Mg, thereby limiting the rate of hydrogen release. Various hydrogen molecule sustained-release and non-sustained-release dressings were applied to the full-thickness skin defect wound model in *C57/BL6* mice. The effects of different hydrogen molecular dressings were analyzed and compared, and the mechanisms under-lying hydrogen’s therapeutic action were explored.

## 2. Materials and Methods

### 2.1. Ethics Statement

All animal studies were conducted in accordance with the protocols approved by the Biomedical Research Ethics Committee of the Sixth Medical Center of PLAGH, China, and all procedures were conducted in accordance with the Regulations on the Administration of Experimental Animal Affairs (China).

### 2.2. Synthesis of Mesoporous Silica-Coated Micro Magnesium and Ammonia Borane

Magnesium powder was obtained from Weihao Magnesium Powder Co. (Tangshan, China). Aminoborane was purchased from Bide Pharmatech Co. (Shanghai, China). Mesoporous silica was obtained from J&K Scientific (Beijing, China). Ammonium hydroxide and acetone were purchased from Huateng Chemical Co., Ltd. (Beijing, China).

Briefly, to create a magnesium micropowder dispersion, 10 mL of 30% ammonium hydroxide was mixed with 162 mL of acetone at room temperature, and the mixture was then stirred for 20 min. Following this, 1 g of magnesium powder was added to the solution and stirred until a uniform mixture was achieved. For the preparation of the mesoporous silica solution, 2 g of mesoporous silica was dissolved in 72 mL of acetone. The solution was gradually added to the ammonium hydroxide and acetone mixture over a period of 2 h, with continuous stirring maintained overnight at room temperature. Subsequently, the mixture was subjected to high-speed centrifugation at 14,000 rpm, and the solid was washed three times with acetone. Finally, the resulting SiO_2_@Mg powder was dried in an electric thermostatic (202-0, Supo Instrument, Shaoxing, China) drying oven at 40 °C.

As for the synthesis of mesoporous silica-coated ammonia borane, mesoporous silica was first prepared by completely dissolving it in distilled water at a mass-to-volume ratio of 10:1. Next, 66 milligrams of ammonia borane were dissolved in 200 microliters of the aforementioned mesoporous silica aqueous solution. The mixture was subjected to high-speed centrifugation at 14,000 rpm for 10 min, and once the supernatant was removed, the precipitated SiO_2_@AB is collected. The collected SiO_2_@AB was then dried using a vacuum freeze dryer (ZLGJ-12, Huachen Kwanda, Zhengzhou, China) and preserved at a temperature of 4 degrees Celsius for storage and future use.

### 2.3. Morphological Analysis of Hydrogen Storage Material

The morphologies of Mg, AB, SiO_2_@Mg, and SiO_2_@AB powder were obtained by SEM (GeminiSEM 300, Carl Zeiss, Shanghai, China). For the preparation of the sample, a layer of conductive adhesive was attached to the sample holder. After that, a toothpick was used to pick out the powder sample and sprinkle it onto the adhesive. Once the sample was securely adhered, an ear syringe was used to blow away any particles that were not firmly attached to the conductive adhesive. The prepared sample was placed in the sample chamber. After vacuuming, the morphology of the sample was observed at magnifications of 15,000×, 100,000×, and 500,000×, respectively.

### 2.4. Release Kinetics of Hydrogen from the Material

The hydrogen storage material sample was immersed in 1 mL of distilled water for 4, 8, 12, 16, and 20 h, respectively. Then, the hydrogen released from the material was determined using gas chromatography–mass spectrometry (GCMS-QP2010 Ultra, SHIMADZU, Kyoto, Japan). For the measurement of the standard curve, hydrogen gas with a purity of 99.999% was generated using a hydrogen generator (ZK-300, Zhongke Huiheng, Beijing, China) and collected in a gas collection bag. Before measurement, the hydrogen was diluted with 99.999% nitrogen gas to prepare standard hydrogen gases of different concentrations. According to the Henry’s Law equilibrium equation, it can be deduced that when the volume of the gas phase in a sealed space is more than twice that of the liquid phase, it can be approximately assumed that all is transferred to the gas phase. The constant linear velocity control method was used, with a constant linear velocity of 60 cm/s, a column temperature of 50 °C, an SH-Rt™-Msieve 5A chromatographic column (Shimadzu, Kyoto, Japan), an injection port temperature of 250 °C, a split ratio of 5:1, a pressure of 200.2 kPa, a column flow rate of 3.85 mL/min, and a BID detector. A 1 milliliter sample was manually injected using the headspace method.

### 2.5. Creation of the Full-Thickness Skin Wound Model

In this study, a full-thickness skin wound model was established. All animal studies were carried out according to protocols approved by the Committee on Ethics of Biomedicine Research, the Sixth Medical Center, PLAGH, China, and all procedures were conducted in accordance with the Regulations for the Administration of Affairs Concerning Experimental Animals (China). Male *C57BL/6N* mice (6 weeks old, 18–20 g) and the necessary supplies including mouse cages, water bottles, mouse feed, and corn cob bedding were purchased from Vital River Laboratory Animal Technology Co., Ltd. (Beijing, China). Animals were maintained under standard conditions at 25 °C with a 12 h light/dark cycle and were fed a normal diet. The mice were acclimatized to the laboratory environment for three days before the creation of the skin wound model.

Prior to surgical procedures, mice were anesthetized with tribromoethyl alcohol (20 mg/mL, 10 μL/g injection) and weighed on a scale. Once anesthetized, the dorsal hair was shaved from the neck to 1 cm above the base of the tail using an animal clipper. Using surgical tweezers to lift the skin along a circle of 1.2 cm in diameter edge, surgical scissors were used to cut the skin along the edge. This then penetrated down to the fascial layer of the mouse skin, thus creating a full-thickness skin defect model. The silicone ring was sutured onto the wound using 0–6 surgical thread and needle, aligned with the neck and tail proportionally on both sides. The remaining unsutured parts of the wound were then secured with approximately 20 stitches. The detailed structure and construction of the wound model is illustrated in the Figure S2.

### 2.6. In Vivo Studies of the Hydrogen Storage Material for Wound Treatment

A wound dressing with a 2 cm outer diameter was created; the dressing consisted of a hollow sponge adhesive attached to an impermeable membrane, and 0.08 g of the hydrogen storage material was added into the hollow of the sponge. The part of the dressing adjacent to the wound was sealed with a waterproof, breathable film and then the sponge adhesive and silicone were sutured together along the circular edge. For the last step, 1 mL of sterilized distilled water was injected into the hollow sponge adhesive containing the hydrogen storage material.

Mice were divided into five groups (*n* = 4) based on different hydrogen storage materials used in the hydrogen molecule dressings: a negative control group with no hydrogen storage material (control) and groups with hydrogen storage materials consisting of magnesium powder (Mg), ammonia borane (AB), mesoporous silica (SiO_2_), mesoporous silica-coated ammonia borane (SiO_2_@Mg), and mesoporous silica-coated ammonia borane (SiO_2_@AB). The surgery time for the mice was designated as Day 0. On Day 2, Day 4, Day 6, Day 8, Day 10, and Day 12, photographs of the mouse wounds were taken, and the hydrogen molecule dressings were replaced. The wound area was quantified using ImageJ (Version 1.8.0). The edges of the wound were outlined and the Analysis function was used to calculate the wound area.

### 2.7. Enzyme-Linked Immunosorbent Assay

On Day 12, whole blood was collected from the samples by using the retro-orbital bleeding method. The whole blood was centrifuged at 5000 rpm for 20 min at a temperature of 4 °C and then the supernatant was carefully aspirated into a sterile cryovial. Blood *bFGF*, *TGF-β*, and *TNF-α* levels were measured by ELISA Kits (meimian), according to the manufacturer’s instructions.

### 2.8. Hematoxylin and Eosin (H&E) and Immunohistochemistry (IHC) Staining

Animals were sacrificed using cervical dislocation on Day 12 and the modeling skin was acquired for hematoxylin–eosin (H&E) staining and the detection of Col-XVII, Collagen-1, α-SMA, Vimentin, Fibronectin, and Integrin-β1 levels in the wound area. Tissues were isolated from the indicated groups, fixed overnight in formaldehyde (4%, *w*/*v*), and embedded in paraffin. And then the tissue was sectioned, de-paraffinized, and prepared for histology. For the H&E staining, following deparaffination and rehydration, the sections were then stained with hematoxylin for 5 min and eosin for 1 min. Immunohistochemistry was performed on formalin-fixed paraffin-embedded tissue with citrate or Tris buffer antigen retrieval with the following antibodies: Col-XVII (Abcam ab16667, 1:100. Cambridge, UK), Collagen-1 (Abcam ab000 1:200), α-SMA (Abcam ab185230, 1:500), Vimentin (Abcam ab47010, 1:250), Fibronectin (Abcam ab125683, 1:500), and Integrin-β1 (Abcam ab68153, 1:500). All sections prepared for IHC staining were blocked in staining buffer containing appropriate control IgG (Goat, Rabbit, etc.). Enzyme-conjugated secondary antibody was applied, followed by visualization using DAB (3,3′-diaminobenzidine, ZSGB-Bio, Shanghai, China). The stained sections were dehydrated through an ascending alcohol series, cleared in xylene, and mounted with a resinous mounting medium. All staining slides were imaged by an upright Olympus microscope (IX71, Olympus, Tokyo, Japan).

### 2.9. Masson Staining

The staining involved treating the sections with Weigert’s hematoxylin for nuclear staining, followed by Biebrich scarlet-acid fuchsin for cytoplasmic staining. Collagen differentiation was achieved by immersing the sections in phosphomolybdic/phosphotungstic acid, followed by staining with aniline blue. After a brief rinse in acetic acid, the sections were dehydrated, cleared, and mounted.

### 2.10. Quantitative Real-Time PCR (RT-qPCR)

Total RNAs were purified from skin tissue using a RNAeasy Mini Kit (Qiagen, Hilden, Germany). An amount of 30 mg of the frozen wound skin tissue stored at −80 °C was taken and the tissue was quickly transferred into a mortar containing liquid nitrogen. Using the pestle, the skin tissue was vigorously ground until the skin tissue turned into a powder. Following the manufacturer’s instructions, the total RNAs of the tissue were acquired. cDNAs were then synthesized in 20 μL reaction mixtures using a cDNA Reverse Transcription kit (NEB), according to standard procedures. Quantitative PCR was performed using a SYBR Green qPCR Kit (Thermo Fisher, Waltham, MA, USA). The primer sequences used were as follows: *WNT1*: F: 5′-TTCGGCAAGATCGTCAACCG-3′, R: 5′-GCCAAAGAGGCGACCAAAATC-3′; *β-Catenin*: F: 5′-CCCAGTCCTTCACGCAAGAG-3′, R:5′-CATCTAGCGTCTCAGGGAACA-3′; *TGF-β1*: F: 5′-CTTCAATACGTCAGACATTCGGG-3′, R: 5′-GTAACGCCAGGAATTGTTGCTA-3′; *Smad2*: F: 5′-ATGTCGTCCATCTTGCCATTC-3′, R: 5′-AACCGTCCTGTTTTCTTTAGCTT-3′; *Notch*: F: 5′-CCCTTGCTCTGCCTAACGC-3′, R: 5′-GGAGTCCTGGCATCGTTGG-3′; *Jaggde1*: F: 5′-ATGCAGAACGTGAATGGAGAG-3′, R: 5′-GCGGGACTGATACTCCTTGAG-3′; collagen-1: F: 5′-GCTCCTCTTAGGGGCCACT-3′, R: 5′-ATTGGGGACCCTTAGGCCAT-3′; *MMP9*: F: 5′-AGACCTGAAAACCTCCAACCTCAC-3′, R: 5′-TGTTATGATGGTCCCACTTGAGGC-3′; *GAPDH*: F: 5′-AGGTCGGTGTGAACGGATTTG-3′, R: 5′-TGTAGACCATGTAGTTGAGGTCA-3′. Relative levels of expression were determined by normalization to *GAPDH* using the ddCt method. The reactions were run in Mx3005P Real-Time qPCR System (Agilent Technology, Santa Clara, CA, USA).

### 2.11. Determination of Alanine and Aspartate Aminotransferase Concentration

The operation steps for alanine aminotransferase and aspartate aminotransferase were the same, and the operations were carried out in a 96-well plate with three replicate wells set for each well. After pre-heating the substrate solution in a 37 °C water bath, 20 μL was added to the measurement wells and control wells, respectively. The mouse tissue homogenate was diluted to 1/100 of its original concentration, and then 5 μL was added to the measurement wells. After aspirating each sample in the measurement wells, the pipette tip was immersed in the substrate solution at the bottom of the well and pipetted up and down repeatedly to mix. Then, the 96-well plate was sealed with a membrane and incubates in a 37 °C water bath for 30 min. Then, 20 μL of 2,4-dinitrophenylhydrazine solution was added to the 96-well plate, and 5 μL of the diluted tissue homogenate was added to the control wells. After aspirating each sample in the control wells, the pipette tip was immersed in the substrate solution at the bottom of the well and pipetted up and down repeatedly to mix. Then, the 96-well plate was sealed with a membrane and incubated in a 37 °C water bath for 20 min. Finally, 200 μL of 0.4 mol/L sodium hydroxide solution was added to the 96-well plate. The 96-well plate was gently shook for mixing, left to stand at room temperature for 15 min, and then the absorbance of each well at 510 nm was measured using a microplate reader. After referring to the standard curve, the corresponding activity units were obtained.

### 2.12. Statistical Analysis

All the data in this experiment were analyzed using GraphPad Prism software (version 8.01). Assuming normality and homogeneity of variances, data were statistically described as mean ± standard deviation (m ± s). For statistical inference, the *t*-test was employed.

## 3. Results

### 3.1. Analysis of Hydrogen Release and Encapsulation Efficiency in Micro-Magnesium and Ammonia Borane Hydrogen Storage Materials

**Figure 1:**
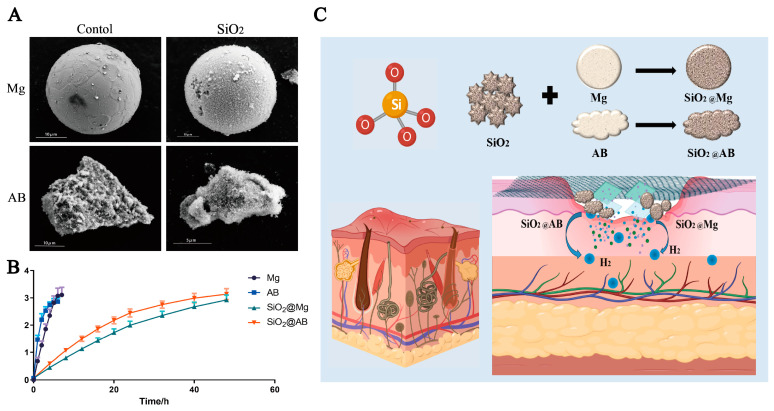
The production and analysis of hydrogen release ability and encapsulation efficiency. (**A**) Scanning electron microscopy (SEM) results show that Mg and AB particles were encapsulated by SiO_2_, respectively. (**B**) Hydrogen production curve of sustained hydrogen release material, tested by gas chromatography standard (GCMS) for 50 h using a GCMS-QP2010 ultra gas chromatography–mass spectrometry system. (**C**) The principle of wound healing with sustain-release hydrogen dressings and a descriptive outline of potential visual illustrations.

Through comparisons of (Figure 1A), the originally smooth surface of the spherical micro-magnesium particles (Mg) was encapsulated with mesoporous silica. The ammonia borane complex (AB) and mesoporous silica (SiO_2_) were uniformly dispersed and combined. The scanning electron microscopy (SEM) results indicate that the hydrogen storage materials were successfully developed into sustained-release hydrogen dressings.

According to the hydrogen release curves of the storage materials shown in (Figure 1B), the AB production curve reached a plateau at the 4th hour, while the Mg production curve reached a plateau at the 6th hour. This suggests that AB releases hydrogen faster than Mg. Additionally, AB produces more hydrogen overall, indicating a stronger hydrogen storage capacity compared to Mg. After encapsulation, SiO_2_@AB reached its plateau phase at the 20th hour, while SiO_2_@Mg reached its plateau at the 40th hour and ceased hydrogen production. This suggests that the sustained-release effect of Mg@SiO_2_ is better than that of AB@SiO_2_. In terms of the total hydrogen production, SiO_2_@AB produced more hydrogen than SiO_2_@Mg, indicating that SiO_2_@AB has a higher hydrogen storage capacity. In summary, encapsulation of the materials slows down the release of hydrogen molecules. Mg@SiO_2_ demonstrated a stronger sustained-release ability after encapsulation, while AB@SiO_2_ exhibited a better overall hydrogen storage capacity. As shown in (Figure 1C), an impermeable membrane prevented the gas from diffusing into the air. Beneath this membrane was a layer of medical sponge rubber, which served as the reaction chamber for the hydrogen release reaction. The system was then sealed with medical protective glue to prevent infection. The non-toxic nature of the materials to mice was demonstrated by detecting the levels of aspartate aminotransferase (AST) and alanine aminotransferase (ALT) after their exposure to these materials in Figure S1.

In our study, the spherical Mg particles resulted from a controlled nucleation–growth synthesis. Gas-phase and wet-chemical methods create a homogeneous environment; in gas-phase synthesis, uniform reactant distribution and weak directional forces enable symmetric growth, and the spherical form minimizes surface energy. Conversely, ammonia borane (AB) synthesis in solution has non-uniform reactant concentrations, rates, and temperatures. Uneven reactant availability causes varied growth rates, and AB’s strong intermolecular hydrogen bonding leads to non-symmetric aggregation, disrupting regular shape formation and causing irregular growth. This accounts for the different shapes of Mg and AB.

### 3.2. The Promoting Effect of Hydrogen Molecule Dressings on Mouse Wound Healing

**Figure 2:**
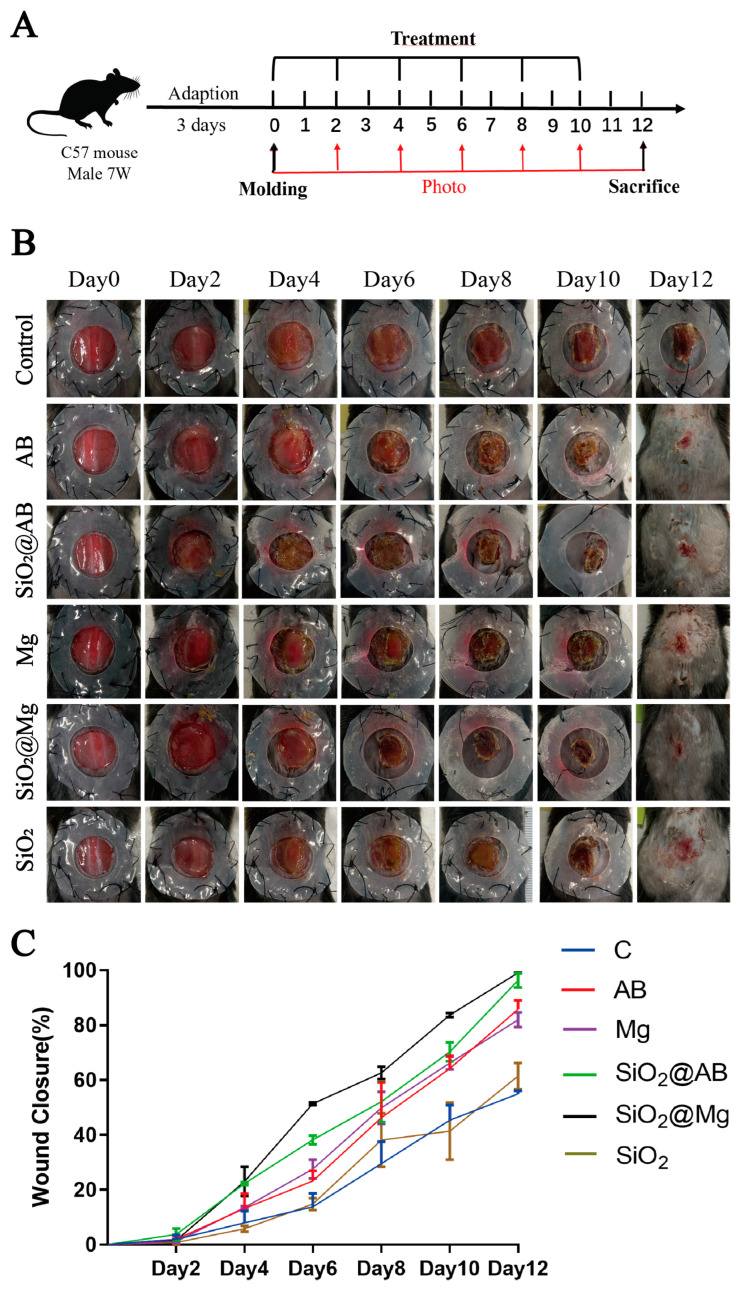
The effect of hydrogen dressings on treating wounds on mouse dorsal skin. (**A**) Animal experiments were performed based on a daily treatment schedule: mice were treated with hydrogen dressing once every two days at 48 h intervals until sacrifice, immediately after modeling. (**B**) Photo of the wound closure status in the five groups during the 12 days healing phase. Control group was treated with blank dressings, and AB, Mg, AB@SiO_2_, Mg@SiO_2_ and SiO_2_ groups treated with corresponding dressings. (**C**) The wound closure ratio of murine wounds in different groups on day 0, 2, 4, 6, 8, 10, and 12. The image scale and size of the wound were measured and calculated using ImageJ software.

This study examined the impact of various types of sustained-release hydrogen dressings on wound healing. Murine skin closely resembles human skin, though it is more wrinkled. To prevent these wrinkles from affecting the wound-healing process, non-harmful materials were used to secure the skin. A circular wound was created to ensure a uniform distance from the edge to the center, allowing for a comprehensive evaluation of the healing rate across the wound. After wound modeling in mice, photographs of the wounds were taken every two days, as shown in (Figure 2A). The scab, a clotted mass of platelets and fibrin, formed on the surface of the mouse wound.

Direct observation of the healing process revealed that the control group started to develop noticeable black scabs on the second day of the inflammatory response, induced by excessive exudation and drying at the wound site. Analysis of the wound-healing ratio, as shown in (Figure 2B) and quantified using ImageJ, revealed differences starting on the sixth day. The wound closure proportion in the SiO_2_@AB and SiO_2_@Mg groups was significantly higher than in the AB and Mg groups. This suggests that the stronger the sustained-release effect of the dressing, the greater the wound-healing rate compared to the control group. These results indicate that hydrogen molecule dressings have a promoting effect on wound healing in mice. The wound-healing results of the SiO_2_ group are similar to the control group. This indicates that the patches containing only SiO_2_ have neither a therapeutic nor an inhibitory effect on wound healing.

A comparison of the effects of the slow-release groups (SiO_2_@AB, SiO_2_@Mg) and fast-release groups (Mg, AB) on wound healing, as shown in (Figure 2C), demonstrate that using mesoporous silica to encapsulate the powders resulted in a difference in wound healing, starting on day 4 (*p* < 0.01). By day 6, the difference was even more pronounced (*p* < 0.0001), with the slow-release material groups achieving a healing ratio of 97% by day 12, significantly higher than the 80% observed in the fast-release hydrogen material groups. In contrast, the SiO_2_@AB group showed differences from the AB group starting on day 4, with significant differences seen on day 6 (*p* < 0.01), but the differences were less pronounced on days 8, 10, and 12. This indicates that sustained-release materials are more effective in promoting healing in a mouse model with a full-thickness skin defect in the dorsal area than fast-release hydrogen materials.

### 3.3. The Impact of Hydrogen Molecule Dressings on Epithelialization in Mouse Wound Healing

**Figure 3:**
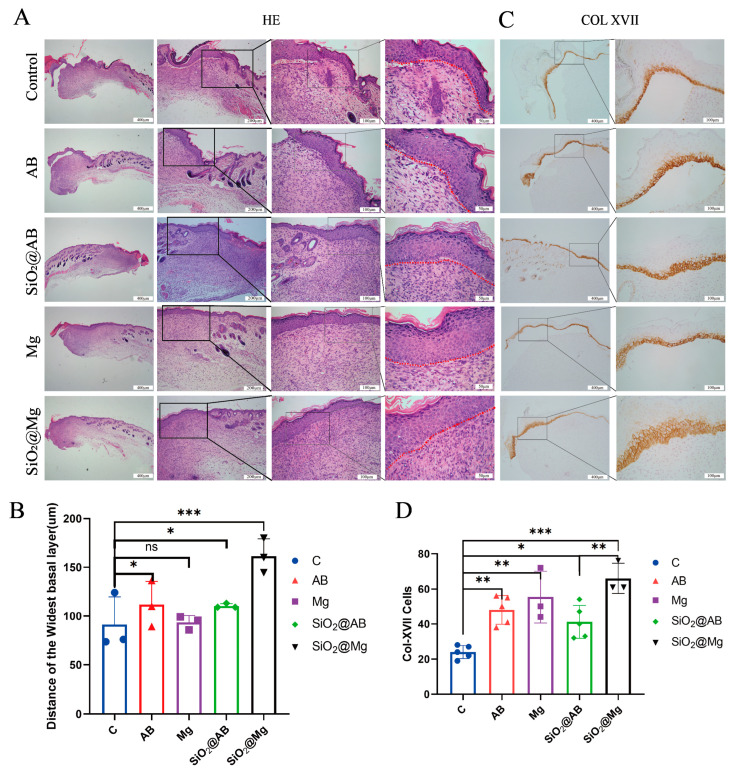
The effect of hydrogen dressings on treating wounds on mouse dorsal skin. (**A**) H&E staining results of control, AB, Mg, AB@SiO_2_, and Mg@SiO_2_ groups. Black box indicates the chosen epidermal area, magnification is indicated on the right. Red dashed line outlines the boundary between epidermis and dermis. (**B**) Statistical analysis of the epidermal thickness in different groups by measuring the distance of the widest basal layer. (**C**) Immunohistochemical staining of Col-XVII. (**D**) Statistical analysis of the Col-XVII expression in different groups by counting the Col-XVII positive cells in area. Scale bar: 400 μm (first and fifth columns), 200 μm (second column), 100 μm (third column), 50 μm (fourth and sixth columns). All data were processed via two-way ANOVA and were plotted as mean ± SEM. *** *p* < 0.001; ** *p* < 0.01; * *p* < 0.05; ns, *p* > 0.05.

Epidermal proliferation and healing begin on the third day after skin injury and continue until the wound is completely closed. To verify the role of hydrogen dressings in this process, hematoxylin and eosin (H&E) staining (Figure 3A), with the red line indicating the boundary between the epidermis and dermis), and ImageJ software were used to measure the thickness of the epidermis near the wound. The results show that all groups experienced epidermal thickening. As shown in Figure 3B, the hydrogen fast-releasing groups (Mg, AB) showed no significant difference in the thickness of the newly formed epidermis near the wound (approximately 100 μm) compared to the control group. However, the sustained-release groups (SiO_2_@AB, SiO_2_@Mg) showed a significant difference (*p* < 0.05), with epidermal thickness around 150 μm, suggesting that the slow-releasing hydrogen dressings have better epidermal-repair capabilities.

Collagen XVII (Col-XVII), a transmembrane protein and a structural component of hemidesmosomes [19], plays a critical role in mediating the adhesion of keratinocytes to the underlying basement membrane [20]. Col-XVII is essential for cell adhesion and regulates the proliferation of keratinocytes during skin regeneration, promoting stable dermo-epidermal adhesion [21]. It is highly elevated near wounds and invasive cancers, accelerating epidermal regeneration (Figure 3C). Studies have shown that keratinocytes lacking Col-XVII exhibit dispersed hemidesmosomal proteins and a loose tissue morphology [22]. The expression of Col-XVII was observed in the new tissue, as shown in Figure 3D. Through direct immunohistochemical staining and counting Col-XVII-positive cells, we found that Col-XVII was primarily located at the interface between the epidermis and dermis. Compared to the negative control group, the number of Col-XVII-positive cells in the hydrogen dressing groups was significantly higher, particularly in the SiO_2_@Mg group. This finding corresponds with the previously observed epidermal layer thickness in each group. These results suggest that hydrogen dressings enhance Col-XVII expression, thereby accelerating epidermal regeneration and promoting wound healing.

### 3.4. The Effect of Hydrogen Dressings on Extracellular Matrix Accumulation in Mouse Wound

The extracellular matrix (ECM) is a non-cellular, three-dimensional macromolecular network, and the extent of ECM accumulation in newly formed skin during the healing process directly impacts the speed and effectiveness of wound closure [23]. During healing, the ECM provides a suitable growth environment for key repair cells, such as endothelial cells, keratinocytes, smooth muscle cells, and fibroblasts [24]. A series of proteins with specific functions, including alpha smooth muscle actin (α-SMA), Vimentin, Fibronectin, and Integrin, work together to regulate ECM accumulation.

**Figure 4:**
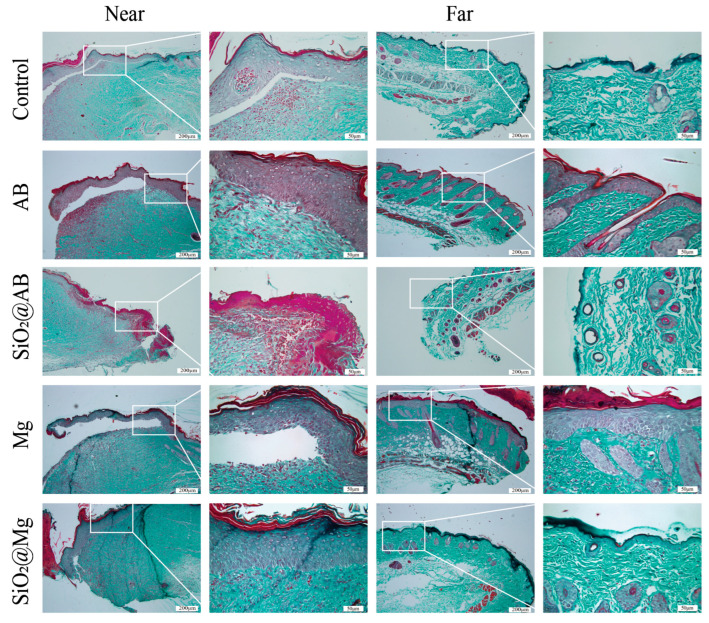
Masson’s staining of both areas near and far from the wounding tissue in a full-thickness skin defect model treated with hydrogen dressing for 12 days. Scale bar: 200 μm (first and third columns), 50 μm (second and fourth columns).

Masson’s trichrome staining is a common method for observing collagen fibers, and the colors are as follows: collagen fibers, mucin, and cartilage are green under the action of anionic dyes; cytoplasm, muscle, cellulose, and neuroglia are red; and nuclei are purple. The results of Masson’s staining, as shown in Figure 4, reveal that in the hydrogen dressing groups, the new wound tissue near the wound edge exhibits more green areas with deep staining, and the collagen fibers are neatly and tightly arranged in a net-like structure. In contrast, the control group shows lighter green staining in the new wound tissue near the wound edge, with sparse and irregularly arranged collagen. These results suggest that treatment with a hydrogen dressing promotes the accumulation and orderly arrangement of collagen fibers in the wound tissue.

### 3.5. The Mechanisms of Extracellular Matrix Accumulation Induced by Hydrogen Dressings in Mouse Wound Healing

The main component of the extracellular matrix (ECM) in the dermis is collagen, which is primarily secreted by fibroblasts during the wound healing process. In the final stages of wound healing, the proportions of different types of collagen begin to change. For example, type III collagen is converted into type I collagen under the action of matrix metalloproteinases (MMPs) to enhance the tissue’s toughness [25]. Type I collagen, a major ECM component, contributes to skin elasticity and toughness while strongly interacting with cells, stabilizing the ECM [26].

To further investigate which type of collagen accumulation is promoted during the healing process, we analyzed the content and distribution of type I collagen (Col-I) using immunohistochemical methods. The positive expression of Col-I in the hydrogen dressing groups was significantly higher than in the control group. The increase in collagen accumulation in the new skin tissue after hydrogen dressing treatment was also reflected in the higher number of positive cells per unit area, indicating that hydrogen dressings promote the accumulation of type I collagen.

In the previous section, we discussed how hydrogen-releasing dressings accelerate wound healing and collagen accumulation. Myofibroblasts, the terminally differentiated form of fibroblasts, play a key role in ECM accumulation and wound healing by generating traction forces on the matrix [27]. Type I collagen is primarily secreted by fibroblasts and myofibroblasts. We speculated that an increase in the number of fibroblasts contributes to both wound healing and Col-I secretion. During wound healing, α-smooth muscle actin (α-SMA) provides the driving force for wound contraction and is a key marker for the differentiation of fibroblasts into myofibroblasts [28]. Therefore, we hypothesized that an increased α-SMA content in tissues promotes myofibroblast differentiation, leading to enhanced collagen accumulation and providing the driving force for wound healing.

**Figure 5:**
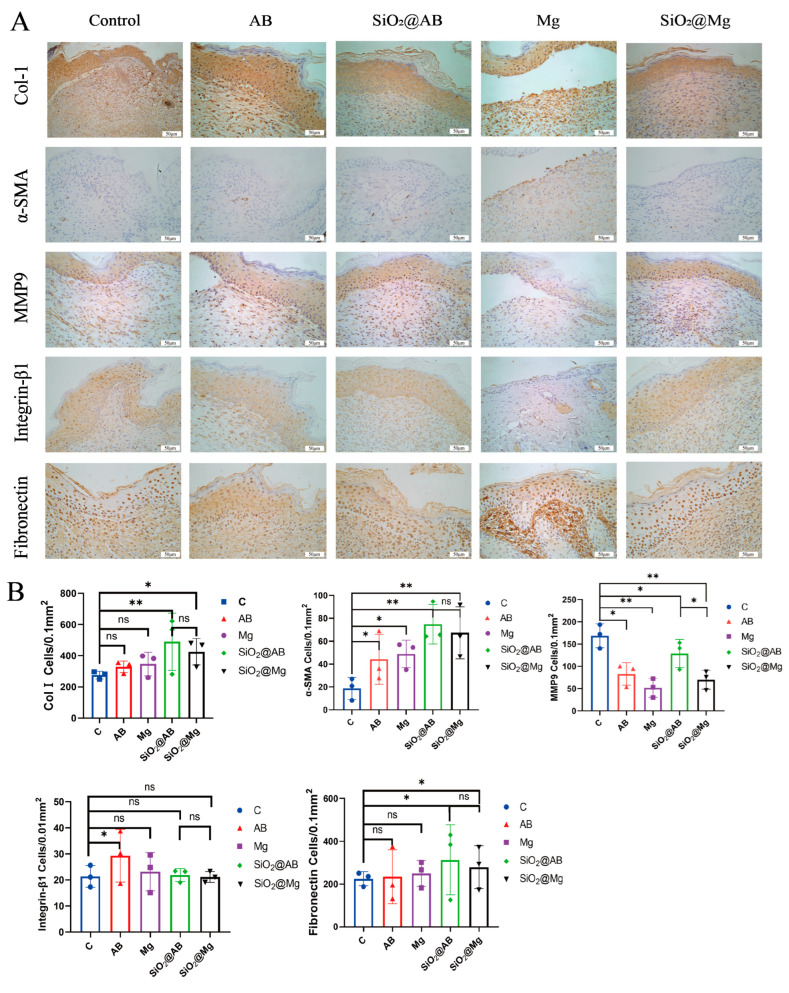
Immunohistochemical staining and analysis of skin tissue in a full-thickness skin defect model treated with hydrogen molecular dressing for 12 days. (**A**) The immunohistochemical staining photos of collagen I, α-SMA, MMP9, Integrin-β1 and Fibronectin (scale bar: 50μm for all images). (**B**) The Prism statistical analysis of the collagen I, α-SMA, MMP9, Integrin-β1, and Fibronectin molecules in different groups of hydrogen dressings. All data were processed via two-way ANOVA and are plotted as mean ± SEM. ** *p* < 0.01; * *p* < 0.05; ns, *p* > 0.05.

To test this hypothesis, we used immunohistochemical methods to detect the expression of α-SMA in new skin tissue during wound healing (Figure 5A). The results indicate that α-SMA-positive cells were primarily located in the proximal region of the newly formed tissue at the wound site. The area of α-SMA-positive cells in the proximal region of the healing wound tissue was significantly greater in the hydrogen dressing groups compared to the control group, although no difference was observed in the distal region of the wound. The number of α-SMA-positive cells per unit area of the healed wound (Figure 5B) revealed a statistically significant difference between the Mg group (rapid hydrogen-releasing group) and the control group (*p* < 0.05). Although the AB group showed a difference compared to the control group, it was not statistically significant. The slow-release hydrogen dressing groups (SiO_2_@AB, SiO_2_@Mg) showed significantly higher expressions of α-SMA than the control group. Furthermore, the increase in α-SMA expression was dependent on the sustained-release effect of the hydrogen molecular dressings, with a stronger sustained-release effect leading to higher α-SMA expression. After 12 days of treatment with hydrogen dressings, the α-SMA content in the proximal region of the newly formed skin tissue was significantly increased. This increase in the α-SMA content reflects enhanced myofibroblast expression, thereby promoting collagen accumulation and validating our previous hypothesis. Moreover, the increased α-SMA content provides a sufficient driving force for tissue repair, thereby shortening the wound healing time.

Matrix metalloproteinases (MMPs) are a large family of enzymes that can degrade nearly all proteins in the extracellular matrix. MMPs are classified into six types based on the substrates they degrade: collagenases, gelatinases, stromelysins, matrilysins, furinactivated MMPs, and other secretory MMPs [29]. MMP9, a collagenase type of MMP, degrades ECM components with the help of zinc or calcium ions, promoting cell migration and angiogenesis. MMP9 is typically expressed at low levels in normal tissues, primarily by neutrophils and macrophages. However, during injury, MMP9 is upregulated to degrade the newly formed ECM. MMP9 targets substrates such as type IV collagen as well as collagen types VII, IX, and X [30]. Excessive MMP9 expression can hinder wound healing by slowing ECM accumulation and wound contraction.

MMP9 expression was highly upregulated in the control group, with positive cells distributed throughout both the proximal and distal regions of the healing tissue. However, in the proximal region of the healing wound tissue, MMP9 expression was significantly lower in the hydrogen dressing groups compared to the control group, with statistically significant differences observed. The Mg group showed a marked difference compared to the control group (*p* < 0.01), and the AB group also exhibited a significant difference (*p* < 0.05). Both the SiO_2_@Mg and SiO_2_@AB groups demonstrated significant differences (*p* < 0.01). Although the SiO_2_@AB group showed a difference from the control group, it was not statistically significant (*p* > 0.05). These results suggest that sustained-release hydrogen dressings can inhibit MMP9 expression in newly formed tissues, preventing ECM degradation, accelerating wound healing, and promoting wound contraction.

Fibronectin (FN), an important chemoattractant, promotes fibroblast migration by binding with fibrin. The integrins and collagens secreted by fibroblasts further stimulate ECM accumulation [31]. To study the effect of hydrogen dressings on FN expression, we detected the number and distribution of FN-positive cells in the newly formed tissue of the wound using immunohistochemistry. FN was distributed abundantly throughout the healing wound tissue, including both the proximal and distal regions. However, there was no significant difference in FN expression between the hydrogen molecule dressing groups and the control group. This indicates that after 12 days of treatment with hydrogen molecule dressings, FN expression during wound tissue healing was not affected. Consequently, hydrogen molecule dressings do not appear to promote wound healing by enhancing ECM accumulation through increased FN expression.

During the later stages of wound healing, Integrin-β1 mediates the interaction between cells and the ECM and transduces physical changes in the ECM into the cells through adhesion molecules, leading to cell proliferation and differentiation [32]. β1-Integrin is expressed in both the proximal and distal regions of the wound, with the highest levels found in the proximal dermis. However, no statistically significant difference in Integrin-β1 expression was observed between the hydrogen molecule dressing groups and the control group. This suggests that the promotion of wound healing by hydrogen molecule dressings is not mediated by changes in Integrin-β1 expression in the skin.

### 3.6. Impact of Hydrogen Dressings on Angiogenesis in Mouse Skin Wound Healing

Angiogenesis, the formation of blood vessels, is a crucial event in the tissue formation phase during skin wound healing. Epithelialization of new tissue depends heavily on an adequate supply of nutrients. A higher number of capillaries and enriched blood flow to the wound site can significantly increase the metabolic rate of the area, reduce capillary fragility, improve tissue oxygenation, and promote wound healing [33]. Following skin injury, the expression of CD31 and vascular endothelial growth factor (VEGF) is activated. CD31, also known as Platelet Endothelial Cell Adhesion Molecule-1, is an integrin protein found on endothelial cells that mediates intercellular adhesion and is closely associated with angiogenesis, serving as a marker for new blood vessels [34]. VEGF is a potent endothelial cell mitogen that stimulates endothelial cell proliferation and promotes the formation of blood vessels [35].

**Figure 6:**
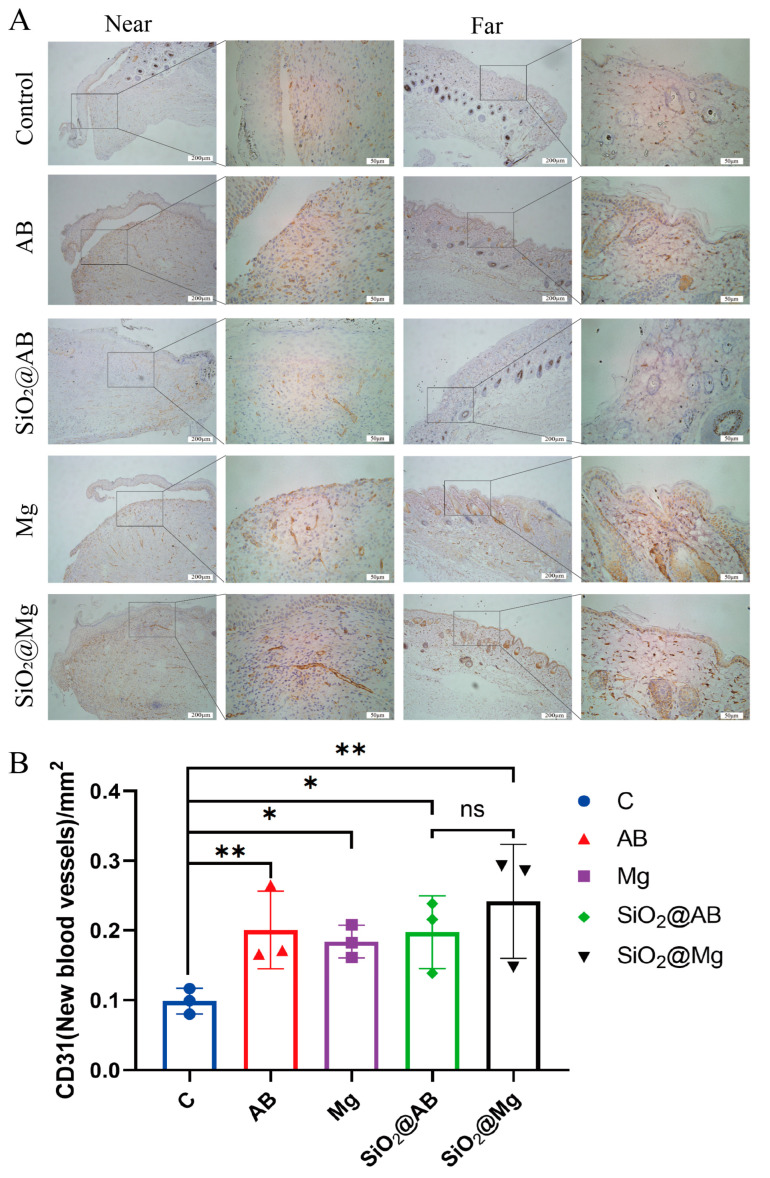
Analysis of angiogenesis in a full-thickness skin defect model treated with hydrogen molecular dressing for 12 days. (**A**) VEGF immunohistochemical staining photos of both near and far areas from the wounding tissue. (**B**) The Prism statistical analysis of CD31 in different groups of hydrogen dressings. Scale bar: 200 μm (first and third columns), 50 μm (second and fourth columns). Data were processed via two-way ANOVA and plotted as mean ± SEM. * *p* < 0.05; ** *p* < 0.01; ns, *p* > 0.05.

To assess the effect of hydrogen dressings on angiogenesis, we performed immunohistochemical staining for VEGF and CD31 in skin tissue sections from various experimental groups. Microvessels were visualized as brownish-yellow to brown-stained areas, appearing as dot-like, tubular, or filamentous structures (Figure 6A).

We quantified the number of blood vessels per unit area near the wound edge (within 2 cm of the wound center). Only brown-stained endothelial cells, not connected to adjacent microvessels or other connective tissue components, were considered as countable microvessels. Immunohistochemical staining for CD31 revealed that both the fast-releasing hydrogen groups (Mg and AB) and the slow-releasing hydrogen groups (SiO_2_@Mg and SiO_2_@AB) exhibited a significantly higher number of positive blood vessels with longer vessel lengths compared to the control group, which showed weak positive staining with shorter vessels. After 12 days of treatment with hydrogen molecule dressings, both the fast- and slow-releasing groups demonstrated a statistically significant increase in the number of blood vessels per unit area compared to the control group (*p* < 0.05) (Figure 6B). This suggests that hydrogen molecule dressings promote angiogenesis in the wound site, which can provide essential nutrients for epithelialization and tissue repair.

Immunohistochemical staining for VEGF in the newly formed tissue of the wound also showed weak positive staining in both the control and hydrogen molecule dressing groups. However, the control group showed almost no positive areas, while the hydrogen molecule dressing groups, particularly those using the slow-release formulations, exhibited significantly higher positivity rates compared to the control group. These findings indicate that hydrogen dressings, particularly those with sustained-release properties, may enhance VEGF expression at the wound site, further supporting the promotion of angiogenesis, and tissue healing indicates the accumulation and orderly arrangement of collagen fibers in the wound tissue.

### 3.7. Investigating the Molecular Mechanism of Wound Healing Promotion by Hydrogen Dressings

The molecular mechanism by which hydrogen molecular dressings promote wound healing remains unclear. To investigate this, we analyzed the expression of several key genes in the newly formed skin tissue of the wound using RT-qPCR.

**Figure 7:**
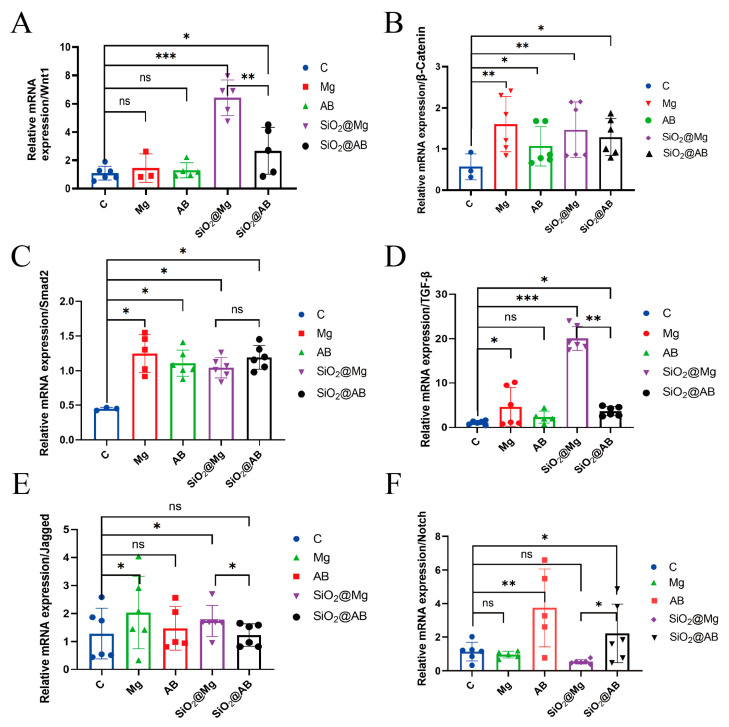
The molecular mechanism of wound healing promotion by hydrogen dressings. *Wnt1* gene (**A**) and *β-catenin* gene (**B**) expression in wound skin tissue. (**C**) *Smad2* gene and (**D**) *TGF-β* gene expression in wound skin tissue. *Jagged1* gene (**E**) and *Notch1* gene (**F**) expression in wound skin tissue. All data were processed via two-way ANOVA and are plotted as mean ± SEM. *** *p* < 0.001; ** *p* < 0.01; * *p* < 0.05; ns, *p* > 0.05.

The *Wnt/β-catenin* signaling pathway plays a crucial role in various cellular activities, including stem cell multipotency, and is involved in skin angiogenesis and epithelial remodeling during wound healing. It is considered one of the key pathways in skin healing. *Wnt1*, a classic upstream gene of the *Wnt/β-catenin* pathway, activates β-catenin by inhibiting the phosphorylation and degradation of glycogen synthase kinase-3β (GSK-3β). This prevents the ubiquitin proteasomal degradation of β-catenin, allowing it to accumulate in the cytoplasm and translocate to the nucleus, where it activates downstream genes. β-catenin promotes the proliferation and differentiation of epidermal cells and fibroblasts, enhances collagen accumulation, and accelerates wound healing [36,37]. The fold change values of *Wnt1* and *β-catenin* gene expression levels are shown in Figure 7A. The expression of the *Wnt1* gene in the SiO_2_@Mg group treated with hydrogen dressings was significantly higher compared to the control group (*p* < 0.005). Figure 7B shows that *β-catenin* gene expression was also significantly increased in the newly formed skin tissue of the hydrogen dressing-treated groups, particularly in the Mg and SiO_2_@Mg groups. Other groups did not show significant increases. These results suggest that hydrogen dressings activate the *Wnt/β-catenin* pathway in the newly formed wound tissues, which promotes the proliferation and differentiation of epidermal cells and fibroblasts, enhances epithelialization, and increases extracellular matrix accumulation, ultimately accelerating wound healing. These findings are consistent with the observed accelerated epithelialization and extracellular matrix accumulation seen in immunohistochemical analyses.

The *TGF-β1/Smad2* signaling pathway is another critical pathway involved in wound healing. As an upstream factor, *TGF-β1* promotes fibroblast differentiation, epithelial tissue formation, and shortens wound healing time. Smad2, a downstream signaling protein of *TGF-β1*, transmits activated signals from *TGF-β1* receptors to the nucleus. It primarily promotes the expression of α-SMA, stabilizes cell structures, accelerates fibrosis, and enhances smooth muscle growth, thus accelerating wound healing [38,39]. Figure 7C,D shows the expression levels of *TGF-β1* and *Smad2* in newly formed wound tissues treated with hydrogen dressings. Specifically, the SiO_2_@Mg and SiO_2_@AB groups (slow hydrogen-releasing groups) showed significantly higher expressions of *TGF-β1* (*p* < 0.005) compared to the control group. Although there was a slight increase in the fast hydrogen-releasing groups, this difference was not statistically significant (*p* > 0.05). Additionally, *Smad2* gene expression was significantly increased in the hydrogen-dressing-treated groups (*p* < 0.05), with no significant difference between fast and slow hydrogen-releasing groups. These results suggest that hydrogen dressings activate the *TGF-β1/Smad2* pathway in wound tissues, promoting α-SMA expression, accelerating fibrosis, and ultimately enhancing wound healing. These findings are in line with previous research.

The *Notch* signaling pathway is highly conserved and regulates gene transcription through interactions between adjacent cells, thereby controlling cell proliferation and differentiation. *Notch* receptors and their ligands are type I membrane proteins [40]. This pathway is involved in the development of various immune cells and regulates MMP9 levels, playing an essential role in maintaining the extracellular matrix [41].

In this study, we examined the expression of *Notch1*, a receptor, and *Jagged1,* a ligand, to assess their role in wound healing. Our results indicated no significant difference in the overall expression of *Notch1* between the hydrogen-dressing-treated groups and the control group (Figure 7E,F). Although the expression of *Notch1* increased slightly in the AB and SiO_2_@AB groups, this difference was not statistically significant. Additionally, no significant changes were observed in the expression of *Jagged1*. These results suggest that hydrogen-releasing dressings do not influence the activation of the *Notch* signaling pathway during wound healing.

## 4. Discussion

While traditional wound dressings such as gauze and cotton pads remain widely employed in clinical practice, they primarily target superficial epidermal layers, largely overlooking the critical physiological role of the dermis in sustaining tissue nourishment and structural integrity. This therapeutic inadequacy significantly compromises the hierarchical wound healing process. Of particular clinical relevance, the formation and remodeling of extracellular matrix (ECM)—especially through regulated collagen fibrillogenesis constitutes a fundamental determinant of successful tissue regeneration [42]. Building on these foundational concepts, our previous investigations uncovered a novel redox-mediated mechanism: molecular hydrogen (H_2_) administration potentiates ECM deposition through the precise modulation of stromal microenvironment dynamics [43]. Mechanistically, we demonstrated that hydrogen exerts temporally specific effects, accelerating the lineage commitment of autologous epidermal stem cells (EpSCs) during the proliferative phase, thereby orchestrating ECM deposition and three-dimensional tissue reorganization. Other studies have also highlighted the role of hydrogen in various skin injury models, such as burn wounds [44,45], pressure ulcers [46], diabetic wounds [47], and radiation-induced dermatitis [48], underscoring its potential in wound healing. Notably, the inherent physicochemical challenges associated with hydrogen gas—particularly its high volatility and low aqueous solubility—create substantial translational barriers to maintaining therapeutic concentrations at wound sites. This pharmacokinetic limitation currently constrains the clinical implementation of hydrogen-based regenerative therapies.

To address this challenge, hydrogen has been integrated with sustained-release materials for wound healing applications. For instance, mesoporous SiO_2_ nanosheets (MSNs) have been developed as a hydrogen-releasing nanomaterial with high biocompatibility. When incorporated into chitosan/hyaluronic acid hydrogel (MSN@CS/HA), they demonstrated significant potential in repairing deeply burned skin [49]. Much of the research on hydrogen therapy for wound healing has focused on key aspects such as macrophage polarization (M1/M2), reactive oxygen species (ROS), and oxidative stress [50]. While numerous studies have emphasized the anti-inflammatory effects of hydrogen and its ability to inhibit bacterial energy metabolism, our study offers novel insights into the sustained-release hydrogen mechanism and its role in promoting collagen accumulation and tissue remodeling.

To enable sustained hydrogen release, researchers have developed diverse hydrogen storage material systems. A representative example involves hydrogen-generating patches that utilize the chemical reaction between calcium hydroxide and aluminum powder, which have demonstrated sustained hydrogen release for periods up to 20 h [51]. Nevertheless, this duration remains inadequate for complete wound healing cycles, compounded by biosafety concerns arising from reaction byproducts including potentially toxic Al^3+^/AlO_2_^−^ ions and alkaline Ca(OH)_2_ residues [52]. To mitigate these limitations, hybrid systems integrating hydrogen storage materials with sustained-release wound dressings have emerged as a promising therapeutic strategy. Hydrogel matrices composed of purified water, carboxymethyl cellulose, and functional additives establish optimal moist wound microenvironments that enhance cellular proliferation [53]. Parallel developments include alginate-based dressings that undergo ion-exchange gelation with sodium ions in wound exudate, effectively maintaining hydration while stimulating tissue regeneration [54,55]. Notably, a symbiotic algae-bacterial biofilm dressing achieved prolonged hydrogen production over 60 h, demonstrating accelerated healing in diabetic wound models [56]. However, the finite viability of microbial consortia in such systems presents implementation challenges, underscoring the need for enhanced biological stability in clinical applications.

Recent advancements employ mesoporous silica nanoparticles (MSNs) as multifunctional carriers for hydrogen storage composites. In a notable advancement, He et al. constructed an MSN@CS/HA hydrogel dressing capable of continuous hydrogen molecule release for approximately seven days under physiological conditions, demonstrating efficacy in burn wound treatment. While this system effectively reduced oxidative stress and promoted neovascularization, it did not fully address extracellular matrix (ECM) regeneration and remodeling—critical processes that influence tissue repair quality.

Both ammonia borane (AB) and magnesium microparticles (Mg) exhibit high hydrogen storage capacities, with mass fractions of 19.6 wt% and 4 wt%, respectively [57]. However, the rapid release of hydrogen from Mg and AB can lead to the formation of hydrogen bubbles, which may pose a potential risk of embolism during tissue repair. To address this issue, it is crucial to integrate hydrogen storage materials with sustained-release systems that enable the gradual release of hydrogen over an extended period. In our recent work, we developed a hydrogen delivery system utilizing mesoporous silica (SiO_2_) to encapsulate AB or Mg. This system facilitates the sustained release of hydrogen, maintaining an optimal concentration at the wound site. This approach not only enhances the efficacy of hydrogen therapy but also improves the quality of wound healing by promoting extracellular matrix (ECM) regeneration and remodeling.

The ECM provides mechanical support to tissues and plays a vital role in their physiological and biochemical functions [58]. Following a skin injury, fibroblasts migrate to the wound site, proliferate, and differentiate to produce collagen, proteoglycans, and other components that constitute the new ECM, thereby facilitating tissue regeneration [59]. Our immunohistochemical analysis revealed a significant upregulation of collagen expression in hydrogen-treated wounds. Specifically, Col-XVII, an essential ECM protein that mediates adhesion between keratinocytes and the underlying basement membrane, plays a critical role in maintaining skin structural stability and regulating keratinocyte migration [60]. Collagen type I (Col-I), a major ECM component, not only enhances skin elasticity and toughness but also promotes ECM stability through strong interactions with cells [61]. Our findings demonstrated that sustained hydrogen release significantly upregulated the expression of both Col-XVII and Col-I, suggesting that hydrogen may stimulate fibroblast proliferation around the wound.

Additionally, hydrogen-treated groups showed a high expression of Vimentin, a marker of epithelial–mesenchymal transition (EMT), indicating that hydrogen release may also promote EMT. The proper concentration of hydrogen at the wound site upregulated the expression and activity of vascular endothelial growth factor (*VEGF*), which, in turn, increased the number of blood vessels around the wound. *VEGF* is a potent mitogen for vascular endothelial cells, stimulating their proliferation and promoting angiogenesis [62]. These results suggest that hydrogen molecular dressings can promote angiogenesis in skin wounds, providing sufficient nutrients for the proliferation and differentiation of skin cells, epithelialization of new tissue, and ECM accumulation.

**Figure 8:**
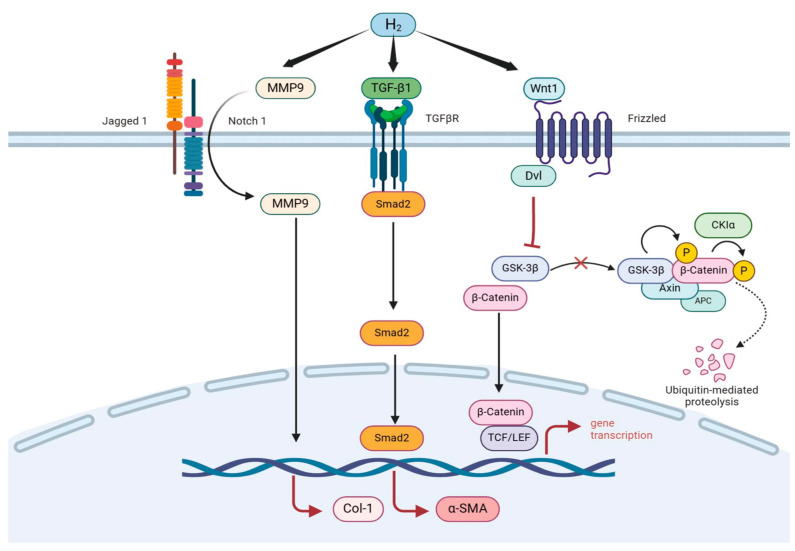
The molecular mechanism of wound healing promotion by hydrogen dressings.

Beyond the morphological and functional observations, we further investigated the molecular mechanisms through which hydrogen-incorporated dressings enhance wound healing as shown in Figure 8. Our analyses reveal a significant activation of both the *Wnt/β-catenin* and *TGF-β1/Smad2* signaling pathways following hydrogen treatment, indicating their pivotal roles in accelerating tissue repair. The *TGF-β1/Smad2* pathway serves as a master regulator of fibroblast differentiation, epithelial tissue regeneration, and wound healing kinetics [63]. As the upstream initiator, *TGF-β1* drives fibroblast differentiation and epithelial tissue formation, thereby reducing the overall healing timeline. The downstream effector *Smad2* transmits signals from *TGF-β1* receptors to the nucleus, where it orchestrates the expression of α-smooth muscle actin (*α-SMA*). This process stabilizes the cytoskeletal architecture, accelerates fibrotic progression, and stimulates smooth muscle proliferation—collectively enhancing wound closure efficiency.

Concurrently, the *Wnt/β-catenin* pathway governs stem cell pluripotency and contributes to cutaneous repair through dual mechanisms: angiogenesis potentiation and epithelial remodeling [64]. The canonical upstream ligand *Wnt1* suppresses glycogen synthase kinase-3β (GSK-3β) activity, thereby blocking β-catenin phosphorylation and proteasomal degradation. Subsequent cytoplasmic β-catenin accumulation facilitates its nuclear translocation, where it activates transcriptional programs for cell proliferation and differentiation. This pathway critically supports dermal regeneration by augmenting collagen deposition and accelerating structural restoration. Notably, matrix metalloproteinase-9 (*MMP9*) emerged as a key modulator of extracellular matrix (ECM) dynamics. While *MMP9*-mediated ECM remodeling is indispensable for wound resolution, its dysregulated overexpression triggers pathological ECM overdegradation, compromising tissue integrity and exacerbating scar formation [65,66]. Our data suggest that hydrogen therapy may fine-tune *MMP9* activity, maintaining its functional output within a therapeutic window to prevent ECM destabilization while preserving physiological repair processes.

In the comparative analysis of hydrogen release performance, SiO_2_@AB demonstrated a superior hydrogen-generation capacity relative to SO_2_@Mg. Paradoxically, the SiO_2_@Mg group exhibited enhanced wound-healing efficacy and more favorable biomarker expression profiles despite its reduced hydrogen output. This apparent discrepancy can be attributed to magnesium’s multifunctional biological roles extending beyond hydrogen production. As an essential micronutrient, magnesium participates in critical biochemical pathways through three primary mechanisms. Magnesium is an essential element in many biological processes [67]. It serves as an important co-factor for DNA polymerases, which play a crucial role in DNA replication and cell proliferation [68]. The presence of magnesium can enhance the activity of these enzymes, thereby promoting cell division and tissue repair at the wound site [69]. Moreover, magnesium is also involved in the activation of various signaling pathways related to cell growth, migration, and inflammation regulation [70,71]. These synergistic effects collectively promote key regenerative processes including enhanced angiogenesis, optimized extracellular matrix remodeling, and controlled inflammation, as evidenced by quantitative biomarker analysis [72]. The therapeutic selection between these systems should be guided by specific clinical objectives: SiO_2_@Mg demonstrates particular advantages for applications requiring rapid epithelialization and collagen synthesis, while SiO_2_@AB may prove more effective in scenarios demanding a precise regulation of matrix metalloproteinase activity. This functional dichotomy underscores the importance of material selection based on targeted physiological requirements rather than singular performance metrics.

This study investigates the therapeutic efficacy of sustained-release hydrogen dressings in wound repair, with particular emphasis on their modulation of collagen remodeling biomarkers and pro-angiogenic signaling pathways. Our findings reveal that molecular hydrogen not only suppresses matrix metalloproteinase-9 (*MMP-9*)-mediated extracellular matrix degradation but also facilitates angiogenesis at the wound periphery. These mechanistic insights establish hydrogen’s dual regulatory capacity in coordinating tissue regeneration processes.

The future application of hydrogen dressings holds considerable promise across a variety of fields. In military settings, for example, hydrogen dressings could be particularly beneficial in treating deep burns and blast injuries by reducing inflammation, preventing infection, and accelerating tissue repair. This would help shorten recovery times and reduce disability rates in soldiers. In clinical practice, hydrogen dressings could be applied to treat a wide range of traumatic injuries, including cuts, lacerations, and abrasions. By promoting wound cleansing, enhancing healing, reducing scarring, and improving overall patient outcomes, hydrogen dressings could significantly enhance the quality of life for patients. For chronic and hard-to-heal wounds, such as diabetic foot ulcers and venous ulcers, hydrogen dressings offer a unique advantage by improving local microcirculation, stimulating angiogenesis, and supplying essential nutrients and oxygen to the wound site, thus accelerating healing. Beyond traditional medical applications, hydrogen dressings may also find new uses in skincare and sports rehabilitation. Their antioxidant and anti-inflammatory properties could be harnessed to improve skin quality and alleviate post-exercise muscle soreness. Advancements in materials science and smart technology may further expand the capabilities of hydrogen dressings. For instance, the development of responsive dressings that automatically adjust the rate and quantity of hydrogen release based on the wound’s condition could optimize treatment outcomes, offering a more personalized and efficient approach to wound care.

In conclusion, hydrogen-based regenerative therapies represent a paradigm shift in wound management and will likely play an increasingly important role in improving human health and well-being.

## Data Availability

Other datasets used and/or analyzed during the current study are available from the corresponding author upon reasonable request.

## Supplementary Materials

The following supporting information can be downloaded at https://www.mdpi.com/article/10.3390/pharmaceutics17030279/s1, Figure S1: Statistical analysis of hepatic aspartate aminotransferase (AST) and alanine aminotransferase (ALT) levels in different groups; Figure S2: The detailed structure and construction method of the model.
