# Supplementary material for: Hydrogen-Releasing Micromaterial Dressings: Promoting Wound Healing by Modulating Extracellular Matrix Accumulation Through Wnt/β-Catenin and TGF-β/Smad Pathways
Source: Pharmaceutics. 2025 Feb 20;17(3):279. doi: 10.3390/pharmaceutics17030279 (PMC11944919; doi:10.3390/pharmaceutics17030279)
Supplement: Supplementary file 1 [file pharmaceutics-17-00279-s001.zip › pharmaceutics-3358970-supplementary.pdf]

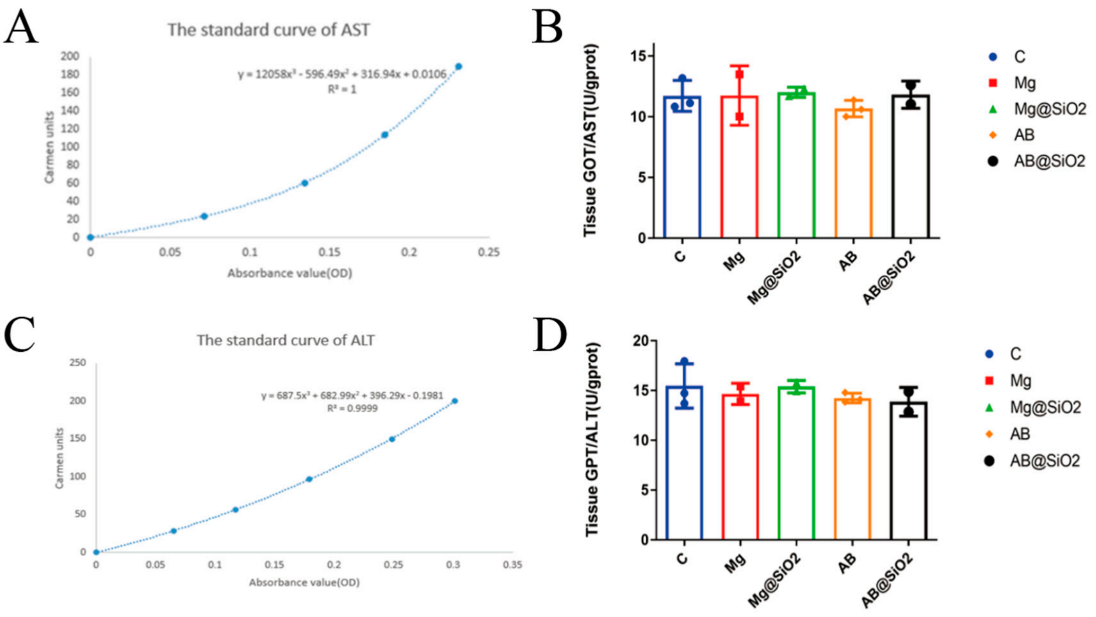

Supplemental Figure S1. Statistical analysis of hepatic Aspartate Aminotransferase (AST) and Alanine Aminotransferase (ALT) levels in different groups. The standard curve of AST (**A**) and ALT (**C**). Contents of aspartate aminotransferase (AST, **B**) and alanine aminotransferase (ALT, **D**) in mouse liver tissue homogenate.

Aspartate aminotransferase (AST) and alanine aminotransferase (ALT) are well - known biomarkers primarily distributed in liver cells. When the liver is exposed to toxins, the integrity of liver cells is compromised, leading to the leakage of AST and ALT into the bloodstream, thus increasing their levels in the serum. As shown in S1, we compared the hepatic AST and ALT levels among the hydrogen - molecule dressing group, and the control group. The results clearly demonstrated that there were no statistically significant differences in the AST and ALT levels between the hydrogen - molecule dressing group and the control group. This consistency in biomarker levels strongly indicates that the application of the hydrogen - molecule dressing to mouse wounds does not affect liver toxicity.

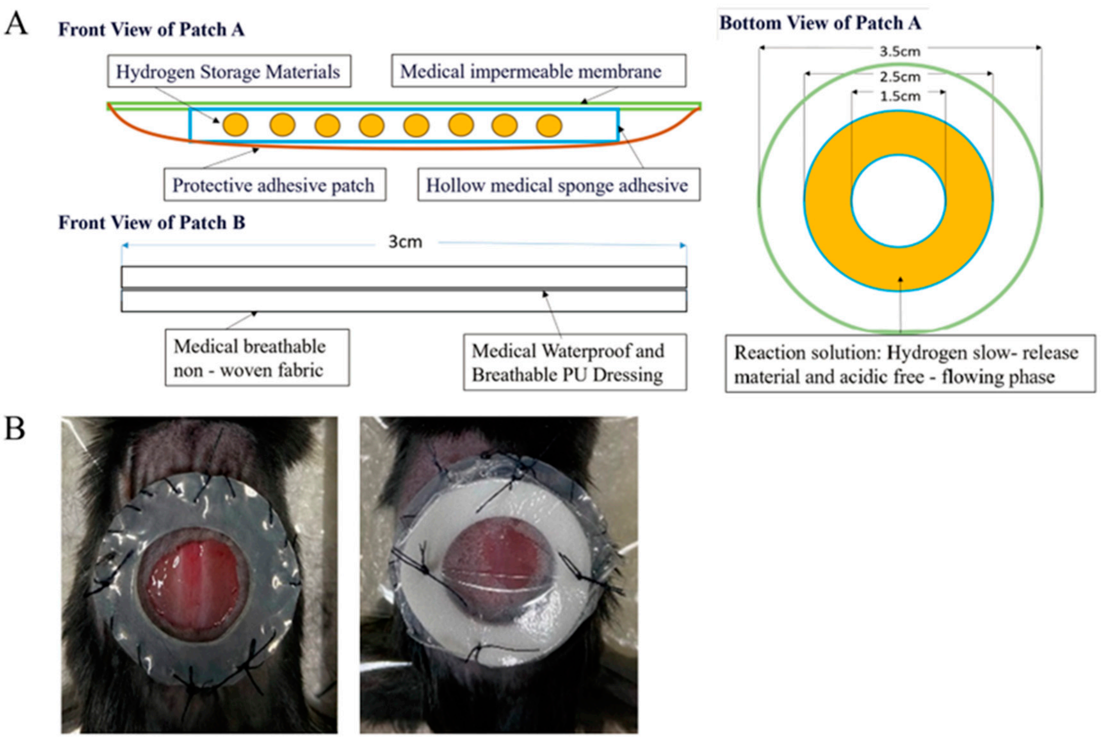

Supplemental Figure S2. The detail structure and construction method of the model. (**A**) Front view of Patch A and patch B (Left), and bottom view of Patch A (Right). (**B**) Schematic diagram of a full-thickness skin defect model on the back of a mouse (Left). Schematic diagram of a model of hydrogen molecular dressing applied to wounds (Right).

The structure of the hydrogen - molecule dressing is divided into two parts: Patch A and Patch B. Patch A serves as the main reaction system, primarily composed of a medical impermeable membrane, a medical sponge adhesive, and a protective adhesive patch. The impermeable membrane, as the layer of the dressing farthest from the skin, functions to prevent gas penetration and diffusion into the air. Beneath the impermeable membrane lies a layer of hollow 3D circular - ring - shaped medical sponge adhesive, which acts as the reaction chamber for the hydrogen - releasing reaction. Subsequently, the sponge adhesive is sealed with a medical protective adhesive patch to prevent external infection. Patch B is mainly composed of a medical waterproof, breathable PU membrane and medical non - woven fabric. Open the medical protective adhesive patch of Patch A, inject an appropriate amount of hydrogen - storage material inside, and then attach the medical PU membrane layer of Patch B to the hollow sponge adhesive and the non - permeable membrane. Attach the medical non - woven fabric layer to the skin in contact with the wound surface, and use a syringe to add deionized water into the reaction chamber through the non - permeable membrane.

Before the experiment, prepare all the reagents. Anesthetize the mice by intraperitoneal injection. Place the mice on a balance to weigh them, and then inject the tribromoethanol anesthetic according to the weight and the proportion described above. After the mice are anesthetized, fix their four limbs and tails to the operating table with tape. Use a depilator to remove the hair on the dorsal skin of the mice. The depilated area ranges from the neck of the mouse to 1 cm above the base of the tail. Dye the edge of a high - temperature - sterilized punch with a diameter of 1.2 cm using a marker pen. Gently press the punch in the middle of the

depilated area on the mouse's back to create a circle with a diameter of 1.2 cm. Then, use high - temperature - sterilized animal surgical forceps to lift the skin along the circular edge, and use surgical scissors to cut the mouse skin along the edge. The surgical depth should reach the fascia layer of the mouse skin, thus establishing a full - thickness skin defect model in C57/BL6 mice. Simultaneously, use surgical sutures with a thickness of 0 - 6 and surgical needles to suture and fix the circular silicone pad to the wound. Place the pad facing the neck, the tail, and at two equally - spaced lateral sides. Then, suture and fix the unfixed parts. The wound model is shown in the figure. Based on the circular defect wound with a diameter of 1.2 cm described above, we fabricated a wound dressing with an outer diameter of 2 cm. The hollow sponge adhesive was adhered to the non - permeable membrane. Then, 0.08 g of hydrogen - storage material was added to the hollow sponge adhesive. The part adjacent to the wound was sealed with a waterproof and breathable membrane. The sponge adhesive and the silicone were sutured together along the circular edge using animal surgical scissors. Subsequently, 1 mL of sterilized distilled water was injected into the hollow sponge adhesive containing the hydrogen - storage material using a 1 - mL syringe. A schematic diagram of the dressing acting on the mouse is provided.

In our system, magnesium reacts with water to form magnesium hydroxide and release hydrogen gas. The reaction equation is:

$$Mg + 2H_2O \rightarrow Mg(OH)_2 + H_2 \uparrow$$

AB reacts with water to produce hydrogen. The reaction can be represented by the simplified equation:

$$NH_3BH_3 + 2H_2O = NH_4^+ + BO_2^- + 3H_2 \uparrow$$

When we add 1 mL of sterilized distilled water into the reaction chamber through the non - permeable membrane, these reactions are triggered, and hydrogen is released to act on the wound area.
